# Kidney dysfunction is associated with mortality, adverse CT-based muscle metrics, and functional decline in surgically treated liposarcomas of the extremities and trunk

**DOI:** 10.1371/journal.pone.0351181

**Published:** 2026-06-15

**Authors:** Julian Kylies, Fabian Haas, Anna Duprée, Tobias B. Huber, Karl-Heinz Frosch, Matthias Priemel, Dominik Kylies

**Affiliations:** 1 Department of Trauma and Orthopedic Surgery, University Medical Center Hamburg-Eppendorf, Hamburg, Germany; 2 Hubertus Wald University Cancer Center Hamburg, University Medical Center Hamburg-Eppendorf, Hamburg, Germany; 3 III. Department of Medicine, University Medical Center Hamburg-Eppendorf, Hamburg, Germany; 4 Hamburg Center for Kidney Health (HCKH), University Medical Center Hamburg-Eppendorf, Hamburg, Germany; 5 Department of General, Visceral and Thoracic Surgery, University Medical Center Hamburg-Eppendorf, Hamburg, Germany; 6 Department of Trauma Surgery, Orthopaedics and Sports Traumatology, BG Klinikum Hamburg, Hamburg, Germany; Stanford University School of Medicine, UNITED STATES OF AMERICA

## Abstract

**Background:**

Liposarcomas (LS) of the extremities and trunk are aggressive soft-tissue sarcomas and surgical resection combined with multimodal therapy represents the cornerstone of curative treatment. Despite advances in surgical and medical management patients are still at risk of developing medical complications that negatively affect morbidity and mortality. Kidney dysfunction, sarcopenia and progressive loss of visceral adipose tissue have emerged as prognostically relevant and potentially treatable complications in surgical oncology. However, despite their growing relevance, little is known about their frequency and impact on survival and morbidity in the context of LS.

**Methods:**

We conducted a retrospective study of 47 adult patients with localized LS of the extremities and trunk who underwent curative-intent surgery. Kidney function, CT morphometry of muscle (skeletal muscle index, SMI) and visceral adipose tissue (VAT) as well as clinical assessments including ECOG score were recorded at diagnosis (t1) and after a median follow-up (t2) of 11 months. Kidney dysfunction, defined as a decrease in eGFR of ≥ 25% between time points, was analyzed in relation to survival, sequentially assessed CT-morphometry of muscle and adipose tissue as well as functional status assessed by ECOG scores.

**Results:**

All patients underwent curative-intent surgical treatment with or without additional multimodal treatment (surgery only: 51.1%, additional radiation: 31.9%, additional chemotherapy: 38.3%). Kidney dysfunction was frequent in our cohort (53.2% of all patients) and significantly associated reduced overall survival in Kaplan–Meier, uni- and multivariate Cox proportional hazards regression models (multivariate hazard ratio: 6.7; p = 0.03). In addition, patients with kidney dysfunction experienced a significantly accelerated loss of SMI (p < 0.001) and VAT (p < 0.001) as well as accelerated functional deterioration measured by worsening ECOG scores between t1 and t2 compared to a stable ECOG score in patients without kidney dysfunction (odds ratio of ECOG increase of: 8.0; p = 0.0027).

**Conclusions:**

To our knowledge, this is among the first studies to investigate kidney dysfunction and its consequences in adult LS patients. In our cohort of surgically treated adult patients with LS of the extremities and trunk, kidney dysfunction was a frequent and clinically impactful complication. It was significantly associated with decreased overall survival, loss of muscle and adipose tissue in sequential CT morphometry assessments and progressive functional decline. Off note, CT-morphometry enabled objective, high-resolution tracking of body composition decline and may serve as a promising additional tool for risk stratification. Nonetheless, given the limited cohort size and retrospective single-center design, the generalizability of our findings is limited and the results should therefore be interpreted with caution. Despite these limitations, our findings call for future prospective studies and an awareness for heightened renal surveillance and integrated body composition assessments in the multimodal management of sarcoma patients.

## Introduction

Liposarcomas (LSs) of the extremities and trunk are aggressive malignant soft-tissue sarcomas [1–4] for which surgical oncologic resection along with multimodal treatment is the mainstay of curative therapy [5–8]. Across all locations, LS account for approximately 13–20% of all soft-tissue sarcomas and are among the most common subtypes [4,9] with an increasing incidence [10], thereby representing an important health burden.

Current guidelines emphasize the importance of multidisciplinary management of LS, involving oncologic surgeons in cooperation with pathologists, medical- and radiation oncologists. In patients eligible for curative treatment, surgical resection represents the cornerstone of therapy, frequently complemented by additional treatment strategies including radiotherapy and/or chemotherapy [5–8], making patients susceptible for a broad range of medical complications.

In this context, kidney dysfunction is a frequent, clinically highly important, treatable but potentially irreversible complication in patients undergoing oncologic surgery that has been linked to morbidity and mortality as well as increased healthcare costs in cancer survivors [11–14]. Moreover, it has been associated with diminished quality of life and an increased risk of treatment-related complications, potentially driven by decreased physiological resilience to chemotherapy, heightened systemic stress, and accelerated catabolic processes [15–21]. Despite its growing recognition as an important risk-factor in oncologic surgery, the frequency and clinical impact remain underexplored in adult patients with LS of the extremities and trunk who may be at particular risk due to extensive surgery and toxic chemotherapy.

Sarcopenia, the progressive loss of skeletal muscle is another important complication in cancer patients that has recently gained growing recognition as a valuable prognostic factor for both complications and survival [22–28]. However, clinical assessment of sarcopenia and adipose tissue, while prognostically relevant, has traditionally been time consuming and user-dependent, limiting its routine application.

In this context, CT-based morphometric analysis has emerged as a valuable prognostic tool, offering objective and standardized measures of muscle mass and body composition. Parameters such as Skeletal Muscle Index (SMI) and Visceral Adipose Tissue (VAT) provide insights into metabolic health and have been increasingly associated with clinical outcomes and survival in oncology settings [29–32].

Interestingly, besides its associations to oncology and surgery [15–19], sarcopenia has also been associated to kidney dysfunction in both experimental and clinical studies, where kidney dysfunction has been shown to mechanistically cause muscle loss via specific molecular cross-talks and was associated with poor outcomes [33–36]. Particularly in surgically treated patients, sarcopenia has been associated with kidney dysfunction and overall mortality [37].

Given its potential impact on clinical outcomes, and to aid improve care in LS patients we here aimed to investigate the frequency and implications of kidney dysfunction on mortality, sarcopenia and loss of visceral adipose tissue measured by CT morphometry as well as declining functional status in surgically treated adult patients with LS of the extremity and trunk.

## Materials and methods

### Ethics statement and patient selection

This retrospective observational study was approved by the Ethics Committee of the Hamburg Medical Association (ethics ID: 2025–300576-WF) and conducted in accordance with the principles of the Declaration of Helsinki. In view of the fact that the patient data that are the subject of the study can no longer be attributed to a human being, the study does not constitute a “research project involving human beings” as defined in Section 9 (2) of the Hamburg Chamber Act for the Medical Professions and also does not fall within the scope of the research projects requiring consultation pursuant to Section 15 (1) of the Professional Code of Conduct for Hamburg Physicians. Therefore, the requirement for informed consent was waived*.*

A systematic screening of our LS database encompassing 108 patients was performed. After employing inclusion and exclusion criteria, a total of 47 patients with histologically confirmed LS of the extremities and trunk who underwent surgical resection between 2010 and 2024 and two available longitudinal CT scans were included.

Inclusion criteria were as follows: age ≥ 18 years, a baseline TNM stage of N0M0, availability of high-quality CT scans capturing the L3 vertebral level at two time points (at diagnosis (t1) and at follow-up (t2)), and complete clinical and laboratory data. Required clinical and laboratory parameters included (at baseline if applicable at follow-up): demographic data, Eastern Cooperative Oncology Group Performance Status (ECOG performance status or ECOG score), resection margin status, TNM classification, tumor diameter, and kidney function assessments at both imaging time points. Patients were excluded if they were under 18 years of age, had metastatic disease at diagnosis, or had missing clinical, laboratory, or imaging data.

Of note, the majority of excluded cases were related to incomplete datasets. As this study aimed to integrate clinical, laboratory, and sequential CT-based morphometric data, we required complete information at both imaging time points (baseline and follow-up). Patients were excluded if CT scans were not of sufficient quality to allow reliable body composition analysis at the L3 vertebral level, or if clinical or laboratory variables were unavailable. Therefore, patients with only a single CT scan, missing follow-up data, or those lost to follow-up were excluded. While this resulted in a reduced final cohort of 47 patients out of a larger initial dataset, the strict inclusion criteria were necessary to ensure internal validity and to allow for robust longitudinal analyses of kidney function and body composition changes. To avoid confounding by nephrectomy-related loss of kidney function, only patients with liposarcomas of the extremities and trunk were included. Retroperitoneal liposarcomas were therefore explicitly excluded from this analysis, as surgical management of these tumors frequently necessitates concomitant nephrectomy, which would introduce a major bias when studying kidney dysfunction incidence and – related outcomes.

### Data collection and definition of outcomes

Clinical and laboratory data were extracted from the electronic medical records corresponding to the two time points of CT imaging (t1 and t2). The first time point (t1) was at initial diagnosis, the second time point (t2) was during follow up after a median of 11 months for the entire cohort. The time intervals between t1 and t2 were similar between the two groups with a median time interval of 11 months (IQR 0 months) in the eGFR loss ≥ 25% group and 11 months (IQR 3,75 months) in the stable group.

Kidney dysfunction was defined as a decline in eGFR of ≥ 25% between t1 and t2 in analogy with previous reports [38,39]. LS patients not meeting the criteria for kidney dysfunction were defined as having a stable kidney function.

**Fig 1:**
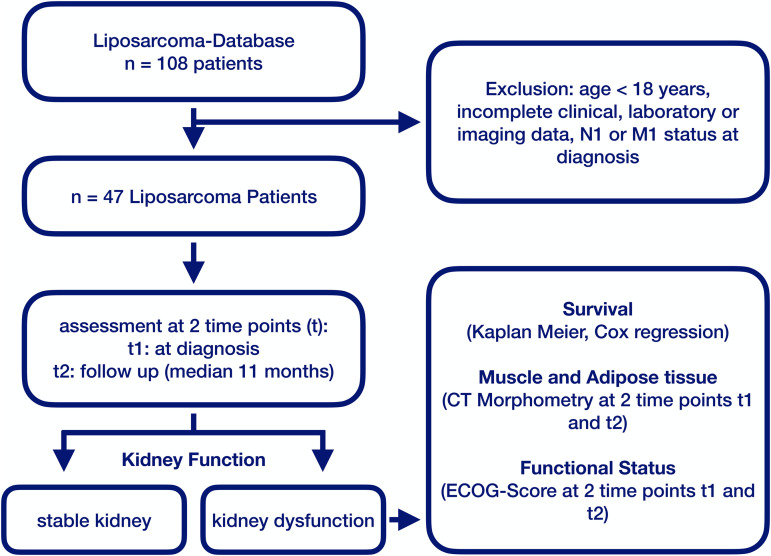
Study design. A screening of our LS database identified 108 LS cases. After employing exclusion criteria (age < 18 years, incomplete clinical, laboratory or imaging data, N1 or M1 status at diagnosis), 47 LS patients were included in the analysis. Assessment of kidney function as well as CT-morphometry and ECOG-Status was performed at diagnosis (t1) and median follow-up of 11 months after diagnosis (t2). Patients were then stratified according to longitudinal kidney function assessment into two groups: those maintaining a stable kidney function and those developing kidney dysfunction. Comparative analysis between these two groups were then performed regarding survival, muscle and adipose tissue mass using CT-morphometry at 2 time points and functional status using the ECOG score at 2 time points (t1 and t2, respectively).

A decline in functional status was defined as having a higher ECOG score at follow-up (t2) compared to baseline (t1). The screening process and study design is illustrated in Fig 1.

Clinical and CT-morphometric data were obtained as of March to June 1, 2025. Survival data were obtained from residents’ registration office as of June 1, 2025. Due to the retrospective and anonymized study design, where there was no access to patient identification, the requirement for informed consent was waived

### Histological tumor grading

Histological grading followed the 2020 WHO Classification of Soft Tissue and Bone Tumors [40], encompassing G1, G2, and G3 liposarcomas. In line with this classification, atypical lipomatous tumors (ALT) of the extremities, which correspond to well-differentiated G1 tumors with essentially benign clinical behavior and negligible metastatic risk, were excluded. By contrast, well-differentiated (G1) liposarcomas located in the trunk were included, together with intermediate- and high-grade tumors (G2–G3).

### Analysis of CT morphometry

CT-based morphometric analysis was performed using Fiji imaging software (Version 2.3.0/1.53q, Max Planck Institute of Molecular Cell Biology and Genetics, Dresden, Germany) with a semi-automated thresholding approach, as previously described [30,41]. All measurements were conducted at the level of the third lumbar vertebra (L3). Skeletal muscle areas were quantified using standardized Hounsfield unit (HU) thresholds (−29 to +150 HU for muscle tissue and −190 to −30 HU for visceral adipose tissue [VAT]) and normalized to patient height (cm²/m²). The assessed morphometric parameters included SMI and VAT.

### Statistical analysis

Statistical analyses and the generation of graphical illustrations were carried out using GraphPad Prism (version 10.4.1) and RStudio (R version 4.4.2). Longitudinal changes in CT-morphometric parameters and if applicable in clinical and laboratory parameters were analyzed using the Wilcoxon matched-pairs signed rank test. Comparisons between patients with and without kidney dysfunction were performed using the Mann–Whitney U test. Survival outcomes were evaluated using Kaplan–Meier curves with Log-Rank (Mantel–Cox) tests and further explored through univariate and multivariate Cox proportional hazards regression models with model comparisons using the Likelihood Ratio Test. Contingency table analysis was performed using the Chi-square test, and odds ratios were calculated to quantify the strength of association. All continuous variables are presented as median with interquartile range (IQR). Statistical tests were two-sided, and p-values below 0.05 were considered indicative of statistical significance.

## Results

### Baseline characteristics

A total of 108 patients were initially screened, of whom 47 patients with complete longitudinal clinical, laboratory, and imaging data at both time points were ultimately included in the final analysis, while the remaining patients were excluded due to missing follow-up data, incomplete datasets, metastatic disease at diagnosis, or age < 18 years. Out of these, 22 (46.8%) LS patients maintained a stable kidney function over the course of the study and 25 (53.2%) developed a kidney dysfunction defined by an eGFR decline of ≥25% between baseline (t1) and follow-up (t2). The median eGFR was 73 (IQR 29.5) and 55 (IQR 21.5) ml/min/1.73 m² at t1 and t2, respectively (p for comparison between t1 and t2 < 0.0001) in the overall cohort. In the stable kidney function group, no significant change in eGFR was observed (median eGFR 69 (IQR 35) and 66 (IQR 37) ml/min/1.73 m² at t1 and t2, respectively, p = 0.84). In the kidney dysfunction group, eGFR declined significantly over the course of the study (median eGFR 76 (IQR 21.5) vs. 45.5 (IQR 11.5) ml/min/1.73 m² at t1 and t2, respectively (p < 0.0001) (S1 Fig). The median age in our cohort was 60.0 (IQR 26.5) years and there was a statistically non-significant trend towards an older age in patients experiencing kidney dysfunction compared to those with a stable kidney function (65.0 (IQR 23.00) vs. 50 (IQR 25.25) years, respectively, p = 0.055). The ECOG performance status score at baseline was 1 (range 2) for all patients without significant differences between patients with stable or worsening kidney function (p = 0.58). Treatment modalities did not significantly differ between groups with comparable portions of patients receiving additional radiation or chemotherapy (p = 0.75 and p = 0.14, respectively). The characteristics at baseline are described in Table 1.

**Table 1:** Baseline characteristics.

	Overall	stable kidney function	kidney dysfunction
**Patients (n)**	47	22	25
**Age (years)**	60.0 (IQR 26.5)	50 (IQR 25.25)	65.0 (IQR 23.00)
**ECOG at baseline**	1 (range 2)	1 (range 2)	1 (range 2)
**Tumor Grade**			
G1 (%)	25.5	27.3	24.0
G2 (%)	31.9	31.8	32.0
G3 (%)	42.6	41.9	44.0
**Surgery only (%)**	51.1	40.9	60.0
**+ Radiation (%)**	31.9	36.4	28.0
**+ Chemotherapy (%)**	38.3	50.0	28.0
**CT-Morphometry**			
SMI (cm^2^/ m^2^)	45.8 (IQR 7.1)	45.6 (IQR 4.8)	47.1 (IQR 8.6)
VAT (cm^2^/ m^2^)	83.4 (IQR 12.9)	82.4 (IQR 20.8)	86.1 (IQR 9.9)

Baseline characteristics of LS patient cohort. Continuous variables if not stated otherwise are presented as median with IQR. Surgery only refers to the fraction of patients treated without additional radiation or chemotherapy. Radiation and Chemotherapy refer to the fraction of patients receiving additional multimodal treatment via radio- or chemotherapy. Some patients received combined radio- and chemotherapy. Minor rounding discrepancies in individual values may occur.

### Impact of declining kidney function on overall survival in LS patients

**Fig 2:**
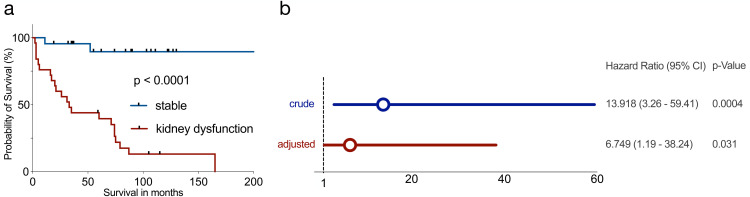
Survival analysis in LS patients stratified by kidney function. **a.** Kaplan-Meier survival analysis shows a significantly decreased survival probability in LS patients experiencing kidney dysfunction compared to patients with a stable kidney function. **b.** Cox proportional hazard regression model analysis. In both the unadjusted (“crude”) as well as the adjusted analysis (adjusted for: age, sex, local recurrence, baseline ECOG, ECOG at follow-up, tumor grade, tumor diameter, wound infections, resection margin status), kidney dysfunction was associated with a significantly increased hazard of death.

To analyze the impact of kidney dysfunction on overall survival we divided our cohort into two groups: LS-patients experiencing a kidney dysfunction and patients who maintained a stable kidney function. First, Kaplan-Meier survival curve analysis demonstrated a significantly worse survival in patients with kidney dysfunction compared to those with a stable kidney function (p < 0.0001, Fig 2a). The corresponding median overall survival was 33.0 months in the kidney dysfunction group, whereas in the stable group the median survival could not be determined as fewer than half of these patients died during the mean follow-up period of 68.5 ± 66.2 months. To further explore the significantly shortened survival in patients experiencing progressive loss of kidney function we generated uni- and multivariate Cox proportional hazards regression models (Tables 2 and 3). The univariate Cox regression confirmed the results of the Kaplan-Meier analysis revealing that a kidney dysfunction was significantly associated with an increased hazard ratio (HR) of death in LS patients (HR: 13.918, 95% CI: 3.260–59.412; p = 0.0004, Fig 2b). In a multivariate model, adjusting for important clinical co-variables (age, sex, local recurrence, baseline ECOG, ECOG at follow-up, tumor grade, tumor diameter, wound infections, resection margin (R-)status), kidney dysfunction remained an independent predictor of decreased overall survival (HR: 6.749; 95% CI: 1.191–38.241; p = 0.031, Fig 2b).

### Muscle and adipose tissue trajectories of LS patients in relation to kidney function

**Fig 3:**
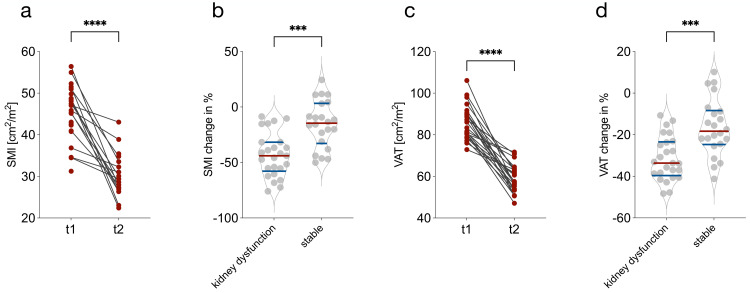
CT-Morphometry of muscle and adipose tissue in LS patients with or without kidney dysfunction. **a.** Longitudinal analysis of SMI-trajectory in patients with kidney dysfunction reveals a significant loss of muscle mass between the time points t1 and t2. **b.** The decline in SMI was significantly larger in patients experiencing a kidney dysfunction compared to those patients with a stable kidney function. **c.** Demonstration of longitudinal VAT-trajectory in kidney dysfunction-patients shows a significant decline in VAT between time points. **d.** This decline in VAT was significantly more accelerated in patients with kidney dysfunction compared to those with a stable kidney function.

We next analyzed muscle and visceral adipose tissue trajectories measured by sequentially assessed CT morphometry in relation to kidney function in LS patients as surrogates of overall body composition, nutritional status, health and resilience. For that, comparable to the survival analysis, we divided patients into two groups (those developing kidney dysfunction and those maintaining a stable kidney function) and then comparatively analyzed the trajectories of SMI and VAT in each subgroup. First and importantly, at baseline (t1) neither CT-morphometry of SMI nor VAT showed significant differences between LS-patients with who maintained a stable kidney function compared to those developing a kidney dysfunction (p = 0.94 for SMI and p = 0.37 for VAT, Table 1). We then assessed the SMI as a quantitative parameter of overall skeletal muscle mass. A longitudinal analysis of SMI in patients experiencing a kidney dysfunction revealed a significant loss of skeletal muscle mass, dropping from a median of 47.05 cm^2^/m^2^ at t1 to 26.99 cm^2^/m^2^ at t2 (p < 0.0001, Fig 3a). When compared to those patients who maintained a stable kidney function across the observation period, this SMI-loss was significantly accelerated in kidney dysfunction LS patients who experienced a loss of −43.9 (IQR 23.6) % compared to only −14.6 (IQR 29.2) % in the stable group (p = 0.0001, Fig 3b). We next analyzed VAT, a quantitative parameter of visceral abdominal fat. Here we found that, similarly to our assessments of muscle mass, VAT also declined significantly between time points t1 and t2 in all patients developing kidney dysfunction: the VAT decreased from 86.07 (IQR 9.89) cm^2^/m^2^ at t1to 57.59 (IQR 9.47) cm^2^/m^2^ at t2 (p < 0.0001, Fig 3c). When comparing the kidney dysfunction to the stable group, the decline in VAT was significantly accelerated in LS-patients experiencing a kidney dysfunction (−33.6 (IQR 15.0) % vs. −18.3 (IQR 14.8) %, p = 0.0002, Fig 3d). Overall, a deterioration of kidney function was therefore significantly associated with a progressive and accelerated loss of skeletal muscle and visceral adipose tissue mass in our cohort, highlighting an accelerated and worsening body composition status in LS patients with kidney dysfunction.

### Associations between kidney dysfunction and functional status

**Fig 4:**
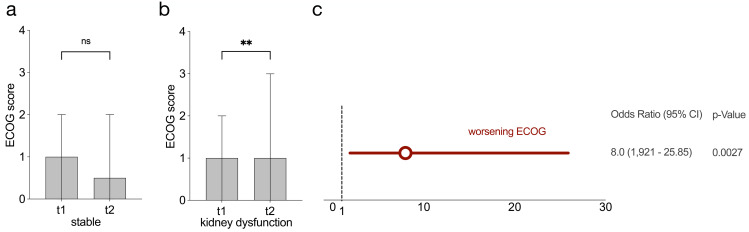
Associations between kidney function and functional status in LS patients. **a.** Longitudinal assessment of ECOG scores. In patients who maintain a stable kidney function, the ECOG score did not significantly change between baseline (t1) and follow up (t2). **b.** Conversely, in patients experiencing a kidney dysfunction, the ECOG score significantly worsened between the two time points. **c.** Forest plot of the odds ratio for declining ECOG score when experiencing a kidney dysfunction.

Ultimately, after linking kidney dysfunction with poor survival and deterioration in body composition measured by CT morphometry, we investigated whether kidney dysfunction was also linked to a decline in functional status. We therefore analyzed the ECOG performance status and its trajectories at both time points in LS patients with and without kidney dysfunction. As noted previously, baseline ECOG performance did not significantly differ between the two groups (p = 0.58, Table 1). Interestingly, in patients maintaining a stable kidney function, the ECOG score did not significantly change over the observation period (median ECOG 1 (range 2) at t1 vs. 0.5 (range 2) at t2, p = 0.3877, Fig 4a). Conversely in patients experiencing a deterioration in kidney function, the ECOG score also deteriorated significantly from a median ECOG 1 (range 2) at t1 to a median ECOG 1 (range 3) at t2 (p = 0.0018, Fig 4b). This was mostly explained because significantly more patients with kidney dysfunction experienced an increase in ECOG score of ≥ 1 category between time points (64% vs 18.2%), corresponding to an odds ratio of ECOG increase of 8.0 (95% CI 1.921–25.85, p = 0.0027, Fig 4c). Overall and similarly to the CT morphometrical assessments of body composition, kidney dysfunction was therefore significantly associated with a decline in functional status as measured by sequentially assessed ECOG scores.

**Table 2:** Univariate Cox proportional hazards regression analysis.

Variable	HR	95% CI	p-value
**Kidney dysfunction**	13.918	3.260 - 59.412	0.0004
**Age**	1.021	0.995 - 1.048	0.117
**Sex**	0.586	0.250 - 1.373	0.218
**Local recurrence**	0.486	0.181 - 1.305	0.152
**Baseline ECOG**	0.781	0.371 - 1.644	0.516
**Second ECOG**	1.672	1.077 - 2.595	0.021
**Tumor grade**	1.001	0.617 - 1.626	0.996
**Tumor diameter**	1.037	0.979 - 1.098	0.213
**Wound infection**	2.545	1.137 - 5.698	0.023
**R-status**	0.751	0.306 - 1.843	0.532

Univariate Cox proportional hazards regression analysis with description of individual model parameters and respective hazard ratios (HR), 95% confidence intervals (95% CI) and p-values.

**Table 3:** Multivariate Cox proportional hazards regression analysis.

Variable	HR	95% CI	p-value
**Kidney dysfunction**	6.749	1.191 - 38.241	0.031
**Age**	1.035	0.985 - 1.087	0.172
**Sex**	0.305	0.071 - 1.313	0.111
**Local recurrence**	0.425	0.105 - 1.718	0.230
**Baseline ECOG**	0.429	0.158 - 1.164	0.097
**Second ECOG**	2.125	1.008 - 4.481	0.048
**Tumor grade**	0.693	0.294 - 1.633	0.401
**Tumor diameter**	1.127	1.032 - 1.232	0.008
**Wound infection**	1.783	0.494 - 6.441	0.377
**R-status**	0.586	0.215 - 1.598	0.296

Multivariate Cox proportional hazards regression analysis with description of individual model parameters and respective hazard ratios (HR), 95% confidence intervals (95% CI) and p-values.

## Discussion

LS is the most prevalent subtype of soft tissue sarcoma in adults, and curative-intent treatment involves surgical resection often complemented by multimodal therapy. Traditionally, oncologic studies in LS have focused on aspects such as local control and histopathological subtype with less attention being paid to additional complications such as declining in kidney function, sarcopenia and loss of adipose tissue [42,43]. However, both kidney dysfunction and sarcopenia, measured by CT morphology, have been increasingly recognized as potentially impactful clinical complications among a spectrum of conditions [44,45]. We therefore conducted a retrospective analysis of LS patients to investigate the frequency and impact of kidney dysfunction on overall survival, sarcopenia and loss of adipose tissue measured by CT morphometry and functional status.

To correctly interpret the results of this study, several methodological aspects and inherent limitations should be considered. In particular, the retrospective longitudinal study design and the requirement for available follow-up imaging and laboratory assessments to classify patients according to kidney function trajectories may have introduced selection bias. As patients with early clinical deterioration, death, or incomplete follow-up data may have been disproportionately excluded, the possibility of survival bias and immortal time bias cannot be excluded and may have influenced the observed survival associations.

Another important methodological aspect of our study is the restriction to extremity and trunk LS. Retroperitoneal LS, while clinically relevant, often require nephrectomy as part of surgical resection, leading to irreversible treatment-related kidney function loss. By excluding retroperitoneal cases, we aimed to eliminate this bias. The retrospective and single-center design in combination with the relatively small size of our cohort, although being comparable to other retrospective studies on liposarcoma and sarcopenia [46], may introduce selection bias and limit the overall generalizability of the results. In addition, relevant clinical comorbidities, including diabetes mellitus, arterial hypertension, and cardiovascular disease, were not systematically assessed in this real-world retrospective cohort, despite their potential influence on both kidney function and overall survival. As these variables were unavailable in a substantial proportion of patients, they could not be reliably incorporated into the statistical analyses, leaving the possibility of residual confounding.

Another potential limitation that arises from the retrospective is that the follow-up imaging was not performed at strictly uniform time points but reflected real-world guideline-based oncologic surveillance intervals. However and importantly, the median follow up between t1 and t2 time was 11 months in both patients with and without declining kidney function and therefore comparable between the subgroups. Another limitation is the use of ECOG performance status which only provides limited information as the sole functional measure: although widely used [47], the ECOG score is a relatively crude tool that may underestimate more subtle changes in quality of life. Here, additional patient-reported outcome measures would potentially yield a more comprehensive assessment of functional decline. In addition, nephrotoxic chemotherapy agents frequently used in sarcoma treatment, including Ifosfamide- and anthracycline-based regimens, may have contributed to kidney dysfunction, systemic toxicity, and progressive muscle wasting in parts of our cohort. However, despite the clinical relevance of this aspect, the relatively small sample size did not allow for a meaningful subgroup analysis of individual chemotherapeutic regimens.

Ultimately, the definition of kidney dysfunction requires discussion: due to the limitations of available data, in particular the limited availability of laboratory kidney assessment in between the defined time points t1 and t2, we chose to define kidney dysfunction as an eGFR decline ≥ 25% between time points in analogy to the frequently used renal outcome parameter “Major Adverse Kidney Event” (MAKE), a measure often used for persistent kidney dysfunction that stretches beyond the relatively narrow time frame of acute kidney injury (AKI) but does not yet fully comply with the definitions of CKD [38,39].

This represents an important limitation of our study our definition of kidney dysfunction based on the the available retrospective data do not allow a reliable distinction between AKI, acute kidney disease, and progression of CKD according to KDIGO concepts. Since kidney function was assessed at two predefined imaging-associated time points separated by a median interval of 11 months, without sufficiently interim laboratory measurements, the observed eGFR decline should be interpreted as an interval-based marker of clinically relevant deterioration in kidney function rather than a definitive diagnostic category. We therefore deliberately used the descriptive term “kidney dysfunction” throughout the manuscript.

Ultimately, another important limitation of our study is the relatively small sample size, which limits both the generalizability of the findings and the statistical power, particularly for multivariable Cox regression models. In the light of these overall limitations, our analysis should therefore be regarded as exploratory and hypothesis-generating rather than definitive.

With these limitations acknowledged, our study nevertheless provides several novel and interesting insights. First, kidney dysfunction was a frequent event in our cohort, affecting more than half of patients. Interestingly, we noticed a trend towards a higher age in patients with declining kidney function that failed to meet statistical significance.

Second, patients who developed kidney dysfunction experienced significantly worse overall survival. Importantly, even after adjusting for important confounders, including age which displayed a trend towards a higher value in the kidney dysfunction group, kidney dysfunction remained an independent prognostic factor. This suggests that it may serve as a clinically meaningful and independent marker of vulnerability in LS patients. Third, kidney dysfunction was linked to progressive body composition decline and functional deterioration, as evidenced by accelerated loss of skeletal muscle and visceral fat and higher rates of ECOG worsening.

The mechanisms underlying these associations are likely multifactorial. Kidney dysfunction is known to promote systemic inflammation, catabolic metabolism, and neurohormonal stress responses, all of which can accelerate sarcopenia [35,48]. An emerging body of evidence now exists describing specific molecular mechanisms by which kidney dysfunction directly and mechanistically promotes muscle loss via the excretion of soluble factors in human patients. Importantly, in experimental models, these factors can already be pharmacologically targeted, preventing these effects [35].

Conversely, low muscle mass may itself predispose to kidney injury by masking renal impairment on creatinine-based assessments and by limiting physiologic reserve. While our study was not designed to determine causality, the observed interactions may suggest a bidirectional relationship between kidney dysfunction and musculoskeletal decline.

In this context, it remains an important consideration is that kidney dysfunction may not only represent a direct contributor to adverse outcomes but could also reflect a broader state of systemic vulnerability and treatment-related toxicity. In particular, chemotherapy-associated nephrotoxicity and catabolic stress may have contributed to declining kidney function, sarcopenia and impaired survival in parts of our cohort. Therefore, causality cannot be inferred from our retrospective observational design, and kidney dysfunction should rather be interpreted as a clinically relevant marker associated with adverse outcomes in this patient population.

From a clinical perspective, these findings highlight the potential of kidney surveillance and body composition monitoring in LS patients undergoing surgical and multimodal curative-intent treatment. Although current sarcoma guidelines do not specifically recommend such measures, our results suggest that they may provide additional information for risk stratification and supportive care. Importantly, we do not propose changes to guideline-based management on the basis of these preliminary data [49,50]. Prospective studies with larger, multicenter cohorts and the integration of patient-reported outcomes will be essential to validate our findings and determine whether incorporating renal and body composition parameters can improve risk-adapted perioperative management in sarcoma surgery.

In conclusion, our study identifies kidney dysfunction as a frequent and clinically relevant complication in surgically treated LS patients, associated with mortality, progressive sarcopenia and functional decline. These results highlight the need for heightened clinical awareness and tailored perioperative management strategies to mitigate the impact of kidney dysfunction in this vulnerable patient population. Interestingly, recent studies have demonstrated that CT-derived renal morphometric features, including kidney volume, cortical thickness, and surface-area-to-volume ratio, are associated with kidney function and CKD severity [51], suggesting that imaging-based renal morphology assessment may represent a promising additional marker of renal vulnerability in future oncologic studies. Future, larger and prospective multicentered studies, incorporating more sensitive measurements (including tighter follow ups, analysis of molecular markers and additional clinical assessment tools such as patient-reported outcome measures, to better assess functional decline) will be essential to validate and expand upon these exploratory yet encouraging findings.

## Supporting information

S1 Fig Longitudinal eGFR trajectories. eGFR at baseline (t1) and follow-up (t2) in **a** all patients, **b** patients with stable kidney function, and **c** patients with kidney dysfunction (defined as an eGFR decline ≥25% between time points).
(PDF)
