# Supplementary figures and images for: Kidney dysfunction is associated with mortality, adverse CT-based muscle metrics, and functional decline in surgically treated liposarcomas of the extremities and trunk
Source: PLoS One. 2026 Jun 15;21(6):e0351181. doi: 10.1371/journal.pone.0351181 (PMC13268190; doi:10.1371/journal.pone.0351181)

a

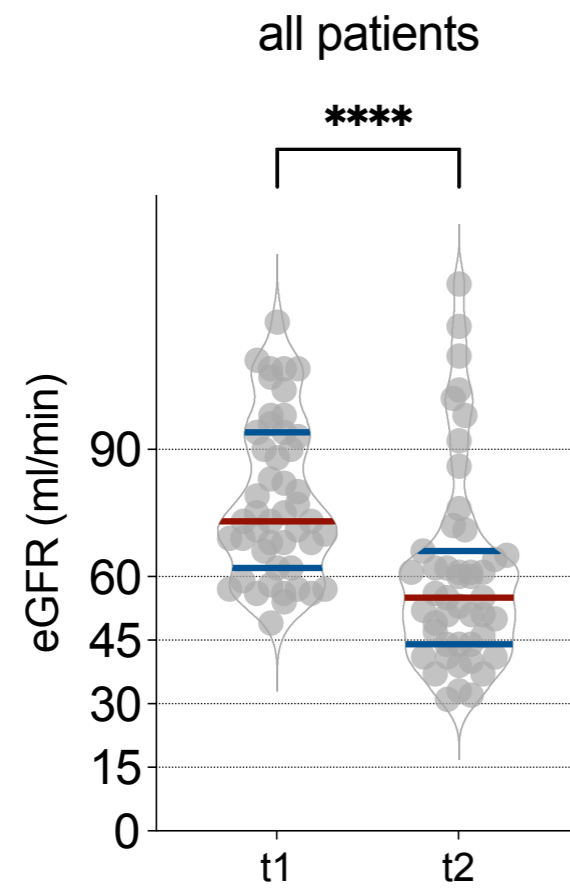

b

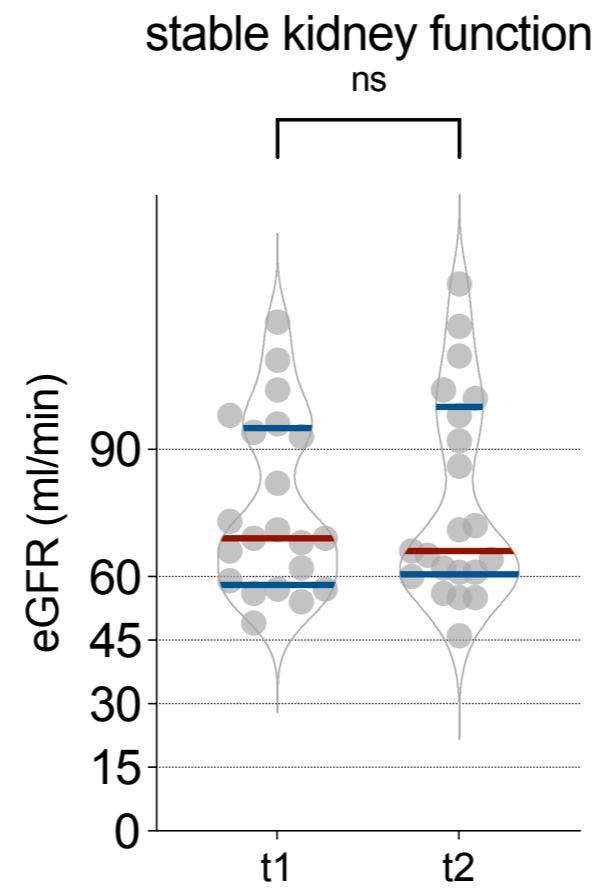

c

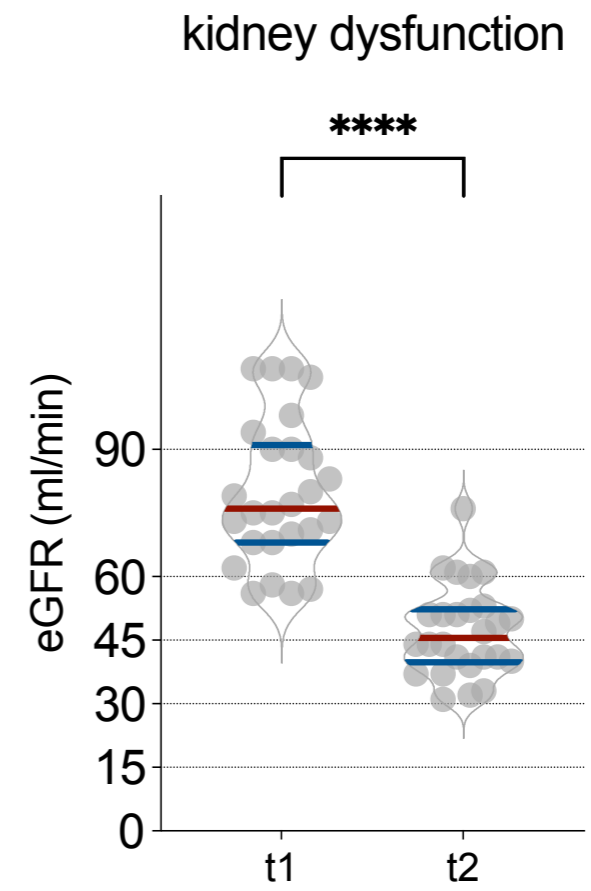

Supplement: S1 Fig — eGFR at baseline (t1) and follow-up (t2) in a all patients, b patients with stable kidney function, and c patients with kidney dysfunction (defined as an eGFR decline ≥25% between time points). (PDF) [file pone.0351181.s001.pdf]
